# Surveillance to Track Progress Toward Polio Eradication — Worldwide, 2019–2020

**DOI:** 10.15585/mmwr.mm7018a2

**Published:** 2021-05-07

**Authors:** Jude N. Tuma, Amanda L. Wilkinson, Ousmane M. Diop, Jaume Jorba, Tracie Gardner, Cynthia J. Snider, Abhijeet Anand, Jamal Ahmed

**Affiliations:** ^1^Polio Eradication Department, World Health Organization, Geneva, Switzerland; ^2^Global Immunization Division, Center for Global Health, CDC; ^3^Division of Viral Diseases, National Center for Immunization and Respiratory Diseases, CDC.

When the Global Polio Eradication Initiative (GPEI) was established in 1988, an estimated 350,000 poliomyelitis cases were reported worldwide. In 2020, 140 wild poliovirus (WPV) cases were confirmed, representing a 99.99% reduction since 1988. WPV type 1 transmission remains endemic in only two countries (Pakistan and Afghanistan), but outbreaks of circulating vaccine-derived poliovirus (cVDPV) occurred in 33 countries during 2019–2020 (*1*,*2*). Poliovirus transmission is detected primarily through syndromic surveillance for acute flaccid paralysis (AFP) among children aged <15 years, with confirmation by laboratory testing of stool specimens. Environmental surveillance supplements AFP surveillance and plays an increasingly important role in detecting poliovirus transmission. Within 2 weeks of COVID-19 being declared a global pandemic (*3*), GPEI recommended continuing surveillance activities with caution and paused all polio supplementary immunization activities (*4*). This report summarizes surveillance performance indicators for 2019 and 2020 in 42 priority countries at high risk for poliovirus transmission and updates previous reports (*5*). In 2020, 48% of priority countries* in the African Region, 90% in the Eastern Mediterranean Region, and 40% in other regions met AFP surveillance performance indicators nationally. The number of priority countries rose from 40 in 2019 to 42 in 2020.^†^ Analysis of 2019–2020 AFP surveillance data from 42 countries at high risk for poliovirus transmission indicates that national and subnational nonpolio AFP rates and stool specimen adequacy declined in many priority countries, particularly in the African Region, suggesting a decline in surveillance sensitivity and quality. The findings in this report can be used to guide improvements to restore a sensitive surveillance system that can track poliovirus transmission and provide evidence of interruption of transmission.

## Acute Flaccid Paralysis Surveillance

Two key performance indicators assess AFP surveillance quality: the nonpolio AFP (NPAFP) rate^§^ and the collection of adequate stool specimens from AFP patients. Based on the background incidence of other acute flaccid paralytic illnesses, an NPAFP rate ≥2 per 100,000 children aged <15 years indicates that a system is sufficiently sensitive to detect circulating poliovirus. Surveillance quality is assured by collection of adequate stool specimens from ≥80% of persons with AFP.^¶^

Surveillance performance in 42 priority countries that had recent WPV or cVDPV transmission or that were deemed at high risk for poliovirus transmission were reviewed. In the World Health Organization (WHO) African Region (AFR), the percentage of priority countries that met targets for both national NPAFP rate and stool adequacy indicators was 67% in 2019 and 48% in 2020 (Table 1). Both surveillance indicator targets were met in 61% of first subnational administrative level areas (e.g., state or province) in 2019 and 53% in 2020 (Figure). Either cVDPV2 cases or environmental isolates were detected in 14 AFR countries in 2019 and in 21 countries in 2020 (Table 1).

**TABLE 1:** National and subnational acute flaccid paralysis surveillance performance indicators and number of confirmed wild poliovirus and circulating vaccine-derived poliovirus cases, by country — 42 priority countries, World Health Organization African, Eastern Mediterranean, European, South-East Asia, and Western Pacific regions, 2019–2020*

Year/WHO region/Country	No. of AFP cases (all ages)	Regional/National NPAFP rate^†^	% Subnational areas with NPAFP rate ≥2^§^	% Regional or national AFP cases with adequate specimens^¶^	% Subnational areas with ≥80% adequate specimens	% Population living in areas meeting both indicators**	No. of confirmed WPV cases	No. of confirmed cVDPV cases^††^
**2019**								
**African Region**	**21,234**	**5.8**	**N/A**	**84.0**	**N/A**	**N/A**	**—** ^§§^	**328**
Angola	578	2.8	66.7	74.4	38.9	14.7	—	138
Benin	310	6.0	100.0	90.6	83.3	84.9	—	8
Burkina Faso	374	4.1	69.2	82.4	84.6	65.6	—	1
Cameroon	613	5.7	80.0	79.8	50.0	35.9	—	—
Central African Republic	230	8.2	100.0	51.7	0.0	0.0	—	21
Chad	821	11.0	95.7	82.8	56.5	68.1	—	11
Congo	195	8.0	100.0	81.0	58.3	61.9	—	—
Côte d'Ivoire	420	3.8	95.0	78.3	50.0	48.0	—	—
Democratic Republic of the Congo	3,808	8.9	100.0	70.6	7.7	7.7	—	88
Eritrea	110	5.0	83.3	100.0	83.3	73.5	—	—
Ethiopia	1,222	2.7	100.0	85.3	90.9	99.6	—	14
Ghana	648	5.2	100.0	87.5	93.8	96.4	—	18
Guinea	233	4.1	100.0	87.6	62.5	59.6	—	—
Guinea-Bissau	44	5.1	100.0	86.4	77.8	70.5	—	—
Kenya	560	2.6	72.3	92.9	78.7	66.9	—	—
Liberia	70	3.3	86.7	91.4	80.0	81.7	—	—
Madagascar	613	5.6	100.0	93.0	86.4	91.9	—	—
Mali	301	3.2	90.9	82.1	63.6	77.8	—	—
Mauritania	55	3.0	86.7	85.5	73.3	59.7	—	—
Mozambique	513	3.6	100.0	72.3	27.3	31.5	—	—
Niger	906	7.7	100.0	67.7	0.0	0.0	—	1
Nigeria	7,509	8.5	100.0	94.1	100.0	100.0	—	18
Senegal	183	2.4	64.3	80.9	57.1	46.7	—	—
Sierra Leone	123	3.7	100.0	78.9	50.0	43.5	—	—
South Sudan	399	7.0	100.0	89.0	90.0	84.0	—	—
Togo	164	4.5	100.0	68.9	50.0	52.2	—	8
Zambia	232	2.8	70.0	81.9	70.0	36.8	—	2
**Eastern Mediterranean Region**	**24,788**	**12.3**	**N/A**	**89.2**	**N/A**	**N/A**	**176**	**26**
Afghanistan	3,768	23.9	100.0	93.8	100.0	100.0	29	—
Egypt	1,343	4.0	92.6	93.4	88.9	85.0	—	—
Iran	1,070	5.5	96.8	97.0	96.8	98.9	—	—
Iraq	1,157	7.1	100.0	94.3	100.0	100.0	—	—
Libya	107	5.9	85.7	98.1	100.0	91.9	—	—
Pakistan	15,218	21.3	100.0	86.6	100.0	100.0	147	22
Somalia	361	5.0	100.0	95.6	100.0	100.0	—	3
Sudan	608	3.6	100.0	96.4	100.0	100.0	—	—
Syria	377	5.8	85.7	85.4	71.4	65.1	—	—
Yemen	779	6.7	100.0	85.8	95.7	97.5	—	1
**European Region**	**226**	**1.7**	**N/A**	**98.2**	**N/A**	**N/A**	**—**	**—**
Tajikistan	92	2.7	100.0	95.7	100.0	100.0	—	—
Uzbekistan	134	1.4	14.3	100.0	100.0	9.5	—	—
**South-East Asia Region**	**420**	**3.0**	**N/A**	**90.2**	**N/A**	**N/A**	**—**	**6**
Burma (Myanmar)^¶¶^	420	3.0	83.3	90.2	83.3	77.0	—	6
**Western Pacific Region**	**1,075**	**2.5**	**N/A**	**51.6**	**N/A**	**N/A**	**—**	**17**
Malaysia	183	2.3	64.3	74.3	42.9	27.9	—	3
Philippines	892	2.5	25.0	47.0	0.0	0.0	—	14
**2020**								
**African Region**	**20,181**	**5.4**	**N/A**	**85.2**	**N/A**	**N/A**	**—**	**532**
Angola	383	2.4	77.8	82.0	61.1	37.3	—	3
Benin	277	5.4	100.0	88.1	91.7	94.5	—	3
Burkina Faso	1,178	11.9	100.0	86.1	92.3	95.2	—	61
Cameroon	605	5.4	100.0	77.9	50.0	40.3	—	7
Central African Republic	222	9.8	100.0	64.4	28.6	28.2	—	4
Chad	990	11.7	95.7	81.4	65.2	69.0	—	99
Congo	93	3.7	66.7	83.9	66.7	31.7	—	2
Côte d'Ivoire	742	6.0	100.0	74.5	39.4	32.6	—	60
Democratic Republic of the Congo	3,303	7.6	100.0	81.0	53.8	55.9	—	81
Eritrea	156	7.0	66.7	99.4	66.7	61.2	—	—
Ethiopia	1,341	2.9	81.8	86.4	81.8	91.5	—	26
Ghana	709	5.9	100.0	85.9	81.2	78.7	—	12
Guinea	321	4.6	100.0	70.1	25.0	16.4	—	44
Guinea-Bissau	21	2.6	45.5	52.4	9.1	13.8	—	—
Kenya	336	1.6	29.8	86.9	70.2	17.4	—	—
Liberia	48	2.3	73.3	95.8	100.0	64.8	—	—
Madagascar	635	5.7	100.0	90.4	95.5	96.4	—	—
Mali	375	3.4	90.9	76.0	45.5	59.9	—	45
Mauritania	17	0.9	26.7	64.7	13.3	0.0	—	—
Mozambique	374	2.6	72.7	73.5	36.4	14.6	—	—
Niger	585	4.8	100.0	72.0	25.0	24.1	—	9
Nigeria	6,330	7.0	97.3	94.5	97.3	97.8	—	8
Senegal	135	1.7	50.0	77.0	28.6	12.2	—	—
Sierra Leone	115	3.2	80.0	78.3	40.0	19.3	—	9
South Sudan	434	6.4	100.0	80.4	70.0	64.3	—	50
Togo	161	4.0	100.0	62.1	0.0	0.0	—	9
Zambia	295	3.6	80.0	69.8	10.0	8.5	—	—
**Eastern Mediterranean Region**	**20,418**	**9.7**	**N/A**	**87.7**	**N/A**	**N/A**	**140**	**546**
Afghanistan	3,972	22.9	100.0	91.9	97.1	98.4	56	308
Egypt	1,009	3.0	85.2	94.5	92.6	93.8	—	—
Iran	618	3.2	87.1	98.5	96.8	91.2	—	—
Iraq	476	2.9	84.2	93.3	94.7	89.0	—	—
Libya	95	5.1	71.4	98.9	100.0	52.9	—	—
Pakistan	11,969	16.4	100.0	85.1	100.0	100.0	84	135
Somalia	378	4.9	85.7	94.2	81.0	94.8	—	14
Sudan	733	3.9	100.0	92.8	94.4	93.6	—	58
Syria	343	5.3	92.9	84.5	78.6	63.6	—	—
Yemen	825	6.8	95.7	77.8	56.5	43.8	—	31
**European Region**	**138**	**1.0**	**N/A**	**95.7**	**N/A**	**N/A**	**—**	**1**
Tajikistan	83	2.4	50.0	92.8	100.0	30.6	—	1
Uzbekistan	55	0.5	0.0	100.0	92.9	0.0	—	—
**South-East Asia Region**	**186**	**1.3**	**N/A**	**85.5**	**N/A**	**N/A**	**—**	**—**
Burma (Myanmar)^¶¶^	186	1.3	22.2	85.5	72.2	9.0	—	—
**Western Pacific Region**	**980**	**2.3**	**N/A**	**65.3**	**N/A**	**N/A**	**—**	**2**
Malaysia	157	2.0	37.5	81.5	62.5	22.9	—	1
Philippines	823	2.4	5.9	62.2	5.9	2.0	—	1

**Abbreviations:** AFP = acute flaccid paralysis; cVDPV = circulating vaccine-derived poliovirus; N/A = not applicable; NPAFP = nonpolio AFP; WHO = World Health Organization; WPV = wild poliovirus.

* Data as of April 16, 2021.

^†^ Per 100,000 persons aged <15 years per year.

^§^ For all subnational areas regardless of population size.

^¶^ Standard WHO target is adequate stool specimen collection from ≥80% of AFP cases, assessed by timeliness and condition. For this analysis, timeliness was defined as two specimens collected ≥24 hours apart (≥1 calendar day in this data set), both within 14 days of paralysis onset. Good condition was defined as arrival of specimens in a WHO-accredited laboratory with reverse cold chain maintained and without leakage or desiccation.

** Percentage of the country’s population living in subnational areas that met both surveillance indicators (NPAFP rates ≥2 per 100,000 persons aged <15 years per year and ≥80% of AFP cases with adequate specimens).

^††^ cVDPV was associated with at least one case of AFP with evidence of community transmission and genetically linked. Guidelines for classification of cVDPV are available. https://polioeradication.org/wp-content/uploads/2016/09/Reporting-and-Classification-of-VDPVs_Aug2016_EN.pdf

^§§^ Dashes indicate that no confirmed cases were found.

^¶¶^ For this country, *MMWR* uses the U.S. State Department short-form name “Burma”; WHO uses “Myanmar.”

**FIGURE:**
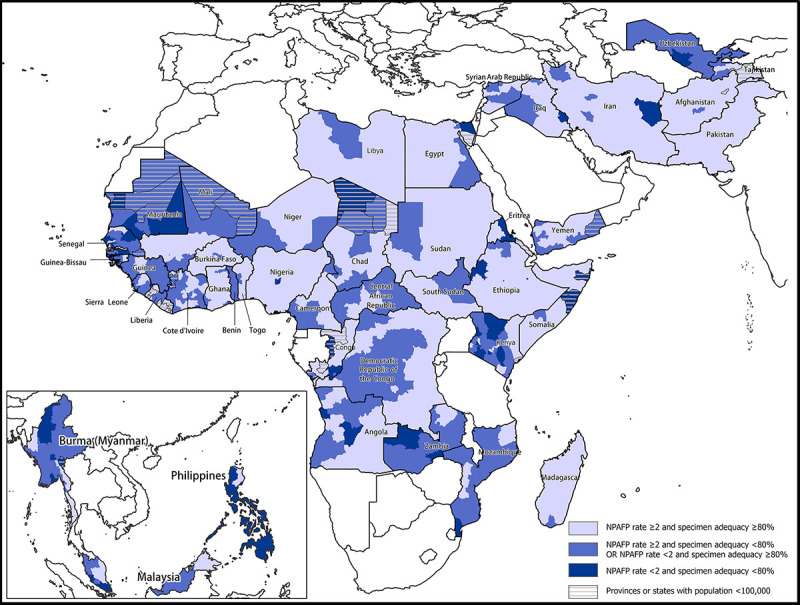
Combined performance indicators for the quality of acute flaccid paralysis surveillance* in subnational areas of 42 priority countries^†^ — World Health Organization African, Eastern Mediterranean, South-East Asia, and Western Pacific regions, 2020 **Abbreviations:** AFP = acute flaccid paralysis; NPAFP = nonpolio AFP; WHO = World Health Organization. * Targets: two or more NPAFP cases per 100,000 children aged <15 years per year and ≥80% of persons with AFP having two stool specimens collected ≥24 hours apart within 14 days of paralysis onset and arrival of these specimens at a WHO-accredited laboratory by reverse cold chain and in good condition. ^†^ For Burma (Myanmar), *MMWR* uses the U.S. State Department short-form name “Burma”; WHO uses “Myanmar.”

All 10 of the assessed priority WHO Eastern Mediterranean Region (EMR) countries met targets for both indicators in 2019, and all but one (Yemen, with stool adequacy of 78%) did so in 2020. Subnational surveillance performance remained high in most EMR countries, but gaps were apparent in Yemen and Libya, where 44% and 53% of the population, respectively, lived in areas that met both surveillance indicator targets in 2020 (Figure). From 2019 to 2020, the number of WPV1 cases declined in the region; cVDPV2 cases increased in Afghanistan (from none to 308), Pakistan (from 22 to 135), Somalia (from three to 14), and Sudan (from none to 58); and in Yemen, significantly more cVDPV1 cases were confirmed in 2020 (31) than in 2019 (one).

In the WHO European Region (EUR), surveillance performance was assessed in the two priority countries of Tajikistan and Uzbekistan. In 2019 and 2020, only Tajikistan met both surveillance indicator targets at the national level. In both years, Uzbekistan met only the stool adequacy indicator target. Subnational surveillance performance was poor in both countries in 2020 (Figure); in Uzbekistan, no subnational area met both surveillance indicator targets. One cVDPV2 case was detected in Tajikistan in 2020, and the subsequent outbreak resulting from this case continues in 2021.

Surveillance performance was assessed in Burma (Myanmar),** the single priority country in the WHO South-East Asia Region (SEAR), where, at the national level, both surveillance indicator targets were met in 2019 and only stool adequacy was met in 2020. Subnational surveillance performance declined from 2019 to 2020; in 2019, 77% of the population lived in areas that met both surveillance indicator targets whereas in 2020 only 9% lived in areas that met both surveillance targets.

In the WHO Western Pacific Region (WPR), surveillance performance was assessed in Malaysia and Philippines. Both countries met the NPAFP indicator target in 2019 and 2020, and neither met the stool adequacy indicator in 2019; however, Malaysia did meet the stool adequacy indicator in 2020. During 2019–2020, approximately one quarter of Malaysia’s population and <3% of Philippines’ population lived in areas that met both indicator targets (Figure); cVDPV1 cases occurred in both countries in 2019, and one case occurred in Malaysia in 2020. Philippines reported cVDPV2 cases in both 2019 and 2020.

Genomic sequence analysis identified 41 cVDPV emergences from AFP cases (39 type 2 cVDPV emergences) in 18 countries in 2019 and 34 cVDPV emergences (32 type 2 cVDPV emergences) in 25 countries in 2020. More than one half (22 of 41) of cVDPV emergences detected in 2019 continued to be detected during 2020.

## Environmental Surveillance

Environmental surveillance is the systematic testing of sewage samples to identify populations shedding polioviruses; environmental surveillance in some locations, might be more sensitive to detection of poliovirus transmission than AFP surveillance, given that paralysis occurs in <1% of poliovirus infections (*6*). During 2019–2020, poliovirus was isolated in a sewage sample before (or in the absence of) a confirmed AFP case in Afghanistan, Cameroon, Chad, Côte d'Ivoire, Egypt, Ghana, Iran, Kenya, Liberia, Senegal (all cVDPV2), Philippines (cVDPV1), and Malaysia (cVDPV1 and cVDPV2).

In Nigeria, the number of cVDPV2 isolations declined from 104 isolates collected from 22 environmental sites in 2019 to 11 isolates collected from three sites in 2020. In Afghanistan and Pakistan, the number of cVDPV2 detections increased from 56 isolates in 2019 (all in Pakistan) to 599 isolates (57% in Afghanistan) resulting from two 2019 cVDPV2 emergences and seven additional new cVDPV2 emergences in 2020.

In 2019, 10 WPV1 genetic clusters (isolates with ≥95% genetic relatedness) were detected in environmental sites from four provinces in Afghanistan and four provinces in Pakistan (*7*). During the reporting period, 30 cVDPV emergences (29 cVDPV2 and one cVDPV1) were detected in sewage samples collected in 26 countries (12 countries in 2019 and 24 countries in 2020).

## Global Polio Laboratory Network

The WHO Global Polio Laboratory Network (GPLN) is an essential component of poliovirus surveillance. It comprises 145 quality-assured poliovirus laboratories in the six WHO regions. GPLN laboratories implement standardized protocols to 1) isolate polioviruses (all laboratories); 2) conduct intratypic differentiation to identify WPV, Sabin (oral polio vaccine) polioviruses, and VDPV (134 laboratories); and 3) conduct genomic sequencing (28 laboratories). Poliovirus transmission pathways are monitored through sequence analysis of the capsid protein (VP1) coding region from isolates. The accuracy and quality of testing at GPLN laboratories are monitored through an annual accreditation program of on-site reviews and proficiency testing (*8*). For laboratories conducting environmental surveillance, another accreditation checklist with separate timeliness indicators is used.

GPLN tested 219,049 stool specimens in 2019 and 147,582 in 2020 (Table 2), and cVDPVs were isolated from 437 AFP cases in 2019 and from 1,067 in 2020. From 2019 to 2020, the number of cVDPV isolates increased from 303 to 530 in AFR, from 50 to 533 in EMR, and from zero to two in EUR; the number decreased from 10 to zero in SEA and from 74 to two in WPR. In 2019 and 2020, all regions met the timeliness indicator for poliovirus isolation.

**TABLE 2:** Number of poliovirus isolates from stool specimens of persons with acute flaccid paralysis and timing of results, by World Health Organization region — worldwide, 2019 and 2020*

WHO region/Year	No. of specimens	No. of poliovirus isolates			% Poliovirus isolation results on time**	% ITD results within 7 days of receipt at laboratory^††^	% ITD results within 60 days of paralysis onset
		Wild^†^	Sabin^§^	cVDPV^¶^			
**African Region**							
2019	51,634	0	1,207	303	93	99	94
2020	47,914	0	3314	530	91	91	NA
**American Region**							
2019	1,957	0	15	0	80	78	88
2020	1,066	0	12	0	81	82	82
**Eastern Mediterranean Region**							
2019	58,924	312	1,927	50	92	99	92
2020	40,179	245	1,311	533	96	61	95
**European Region**							
2019	3,295	0	52	0	83	100	87
2020	2,016	0	24	2	89	73	82
**South-East Asia Region**							
2019	88,734	0	1,807	10	94	98	97
2020	44,799	0	1,315	0	94	95	90
**Western Pacific Region**							
2019	14,505	0	164	74	97	96	71
2020	11,608	0	124	2	96	100	84
**Total^§§^**							
**2019**	**219,049**	**312**	**5,172**	**437**	**95**	**99**	**96**
**2020**	**147,582**	**245**	**6,100**	**1,067**	**94**	**84**	**92**

**Abbreviations:** AFP = acute flaccid paralysis; cVDPV = circulating vaccine-derived poliovirus; ITD = intratypic differentiation; NA = not available; WHO = World Health Organization.

* 2019 data as of March 18, 2020; 2020 data as of March 25, 2021.

^†^ Number of AFP cases with WPV isolates.

^§^ Either 1) concordant Sabin-like results in ITD test and VDPV screening, or 2) ≤1% VP1 nucleotide sequence difference compared with Sabin vaccine virus (≤0.6% for type 2).

^¶^ For poliovirus types 1 and 3, 10 or more VP1 nucleotide differences from the respective poliovirus; for poliovirus type 2, six or more VP1 nucleotide differences from Sabin type 2 poliovirus.

** Results reported within 14 days of receipt of specimen.

^††^ Results of ITD reported within 7 days of receipt of specimen.

^§§^ For the last three indicators, total represents weighted mean percentage of regional performance.

The South Asia genotype (the only WPV1 genotype detected globally since 2016) was detected in Afghanistan and Pakistan in 2019 (176 cases) and 2020 (140 cases). Orphan isolates (those with ≤98.5% genetic identity in VP1, compared with other isolates) indicate possible gaps in AFP surveillance; in 2019, orphan isolates accounted for five of 176 (3%) WPV1 isolates from AFP patients (two in Afghanistan and three in Pakistan) and in 2020 for 18 of 140 (13%) (11 in Afghanistan and seven in Pakistan).

## Discussion

From 2019 to 2020, national NPAFP rates and stool adequacy declined overall in priority countries; subnational surveillance performance declined overall except for WPR countries. Although the total number of WPV1 cases decreased globally from 2019 to 2020, the increase in orphan WPV1 isolates between 2019 and 2020 in both countries suggests gaps in AFP surveillance. The COVID-19 pandemic substantially affected polio eradication activities in 2020 (*9*). In most AFR countries, polio surveillance field and laboratory staff were reemployed to support COVID-19 response efforts as recommended by GPEI (*4*). Surveillance staff and GPEI logistical assets supported COVID-19 surveillance, contact tracing, and data management. The virologic analyses of COVID-19 specimens increased the workload of GPLN staff, who often analyze specimens from multiple laboratory networks. During 2020, movement restrictions in many countries led to batching stool specimens and sewage samples before shipping to the national level (*9*). For countries with no internal WHO-accredited national polio laboratories, transport was further impeded by international travel restrictions.

The findings in this report are subject to at least three limitations. First, factors including security concerns and hard-to-reach subpopulations could affect national and subnational AFP surveillance indicators and limit their interpretation. Second, high NPAFP rates do not necessarily indicate highly sensitive surveillance because some reported AFP cases might not meet the case definition, some actual AFP cases might go undetected, and apparent adequate national data can obscure wide heterogeneity in subnational AFP rates. Finally, the accuracy of stool specimen collection timeliness depends on whether the field investigator can elicit an accurate paralysis onset date.

Sensitive AFP surveillance is critical to detecting poliovirus transmission and relies on timely case detection, notification, investigation, specimen transport, and laboratory testing. With adherence to proper infection control precautions, activities to restore sensitive surveillance must be pursued. Given the successful repurposing of polio resources to support COVID-19 pandemic challenges, further investments in disease surveillance could enable the program to respond to new threats. Thoughtful and planned action is needed as country Expanded Programmes on Immunization move to integrate surveillance for vaccine-preventable and other diseases.

**Summary**

**What is already known about this topic?**

Global polio eradication relies on detecting poliovirus transmission, primarily through acute flaccid paralysis (AFP) surveillance supplemented by environmental surveillance of sewage samples.

**What is added by this report?**

Analysis of 2019–2020 AFP surveillance data from 42 countries at high risk for poliovirus transmission indicated that national and subnational nonpolio AFP rates and stool specimen adequacy declined in many priority countries.

**What are the implications for public health practice?**

The findings provided in this report can help guide improvement efforts to restore timely and sensitive field surveillance activities, which were adversely affected by the COVID-19 pandemic.

## Footnotes

* 2020 priority countries: *African Region*: Angola, Benin, Burkina Faso, Cameroon, Central African Republic, Chad, Congo, Côte d’Ivoire, Democratic Republic of the Congo, Eritrea, Ethiopia, Ghana, Guinea, Kenya, Liberia, Madagascar, Mali, Mauritania, Mozambique, Niger, Nigeria, Senegal, Sierra Leone, South Sudan, Togo, and Zambia; *Eastern Mediterranean Region*: Afghanistan, Egypt, Iran, Iraq, Libya, Pakistan, Somalia, Sudan, Syria, and Yemen; *European Region*: Tajikistan and Uzbekistan; *South-East Asia Region*: Burma (Myanmar); *Western Pacific Region*: Malaysia and Philippines.

† Countries for this report (2019–2020) were selected according to whether they had endemic transmission, had ≥1 cVDPV isolate from AFP or environmental surveillance, or were deemed to be programmatically at high risk.

§ The number of NPAFP cases per 100,000 children aged <15 years.

¶ Two stool specimens collected ≥24 hours apart and within 14 days of paralysis onset, and arrival at a WHO-accredited laboratory by reverse cold chain (storing and transporting samples at recommended temperatures from the point of collection to the laboratory) and in good condition (i.e., without leakage or desiccation).

** For this country, MMWR uses the U.S. State Department short-form name “Burma”; WHO uses “Myanmar.”
